# Activator-induced conformational changes regulate division-associated peptidoglycan amidases

**DOI:** 10.1073/pnas.2302580120

**Published:** 2023-06-05

**Authors:** Jonathan Cook, Tyler C. Baverstock, Martin B. L. McAndrew, David I. Roper, Phillip J. Stansfeld, Allister Crow

**Affiliations:** ^a^School of Life Sciences, University of Warwick, Coventry CV4 7AL, United Kingdom

**Keywords:** bacterial cell division, X-ray crystallography, peptidoglycan, structural microbiology, Amidases

## Abstract

AmiA and AmiB are peptidoglycan-hydrolyzing enzymes from *Escherichia coli* that are required to break the peptidoglycan layer during bacterial cell division and maintain integrity of the cell envelope. In vivo, the activity of AmiA and AmiB is tightly controlled through their interactions with the membrane-bound FtsEX–EnvC complex. Activation of AmiA and AmiB requires access to a groove in the amidase-activating LytM domain of EnvC which is gated by ATP-driven conformational changes in FtsEX–EnvC complex. Here, we present a high-resolution structure of the isolated AmiA protein, confirming that it is autoinhibited in the same manner as AmiB and AmiC, and a complex of the AmiB enzymatic domain bound to the activating EnvC LytM domain. In isolation, the active site of AmiA is blocked by an autoinhibitory helix that binds directly to the catalytic zinc and fills the volume expected to accommodate peptidoglycan binding. In the complex, binding of the EnvC LytM domain induces a conformational change that displaces the amidase autoinhibitory helix and reorganizes the active site for activity. Our structures, together with complementary mutagenesis work, defines the conformational changes required to activate AmiA and/or AmiB through their interaction with their cognate activator EnvC.

## Significance

Peptidoglycan amidases break the peptidoglycan layer during cell division and maintain integrity of the cell envelope. Here, we present structures of an isolated peptidoglycan hydrolase in an autoinhibited (“off”) state and a second amidase bound to the activating LytM domain of EnvC revealing the active (“on”) state. A comparison of these structures provides important molecular insights into the activation of peptidoglycan hydrolases by their cognate activators.

The peptidoglycan layer is a complex molecular mesh that surrounds the bacterial cytoplasmic membrane, providing structural rigidity and protection from osmotic shock (1). In gram-negative bacteria, the peptidoglycan layer also serves as a point of attachment for the outer membrane and defines their characteristic shapes (2). During cell division, the peptidoglycan layer is broken at the division septum to allow insertion of new peptidoglycan and to separate daughter cells. In *Escherichia coli* (*E. coli*), splitting the peptidoglycan layer at the division site involves activity of three closely related periplasmic peptidoglycan amidases (3). AmiA is the smallest division-associated amidase, consisting of a simple zinc-dependent enzymatic domain of ~28 kDa. AmiB and AmiC are larger amidases (45 and 43 kDa, respectively), composed of an AmiA-like enzymatic domain and a small N-terminal domain (the “amiN” domain) that is suspected to be important in anchoring these proteins to the peptidoglycan layer (4, 5) and localization to the division site (6, 7). Both AmiA and AmiC are directed to the periplasm by the twin arginine repeat translocation (TAT) system while AmiB is exported by the Sec pathway (6, 8). A fourth amidase, AmiD, is a membrane-anchored lipoprotein that is not involved in cell division and belongs to a structurally distinct family of zinc-dependent amidases (9).

All the three division-associated amidases have overlapping enzymatic functions in hydrolyzing the peptidoglycan amide bond between the sugar and the first amino acid of the peptide cross-link (3). Single-gene knockouts of *amiA*, *amiB*, and *amiC* each have modest cell separation defects; however, strains lacking multiple amidases have severe chaining phenotypes (3). Strains lacking amidases also have increased sensitivity to antibiotics and detergents, suggesting envelope defects that allow penetration of molecules that would not usually cross the outer membrane barrier (3, 8, 10).

Because of the importance of the peptidoglycan layer for bacterial viability and cell envelope integrity, the activation of peptidoglycan amidases is carefully controlled to guard against lysis or exposure to noxious compounds in the environment. In their resting states, zinc-dependent peptidoglycan amidases such as AmiB and AmiC adopt autoinhibited conformations in which their active sites are blocked by an alpha helix containing a conserved glutamate residue which binds to the active-site zinc (5, 11). Activation of amidases is then stimulated by proteins that bind to the amidases to promote enzymatic activity (11–13). In *E. coli*, several amidase “activator” proteins (also known as murein hydrolase activators) have been identified including ActS (14, 15), EnvC (13, 16), and NlpD (13). The activators share a common motif, the LytM domain (7, 12), which forms the proposed site of amidase binding and activation (7, 17). The LytM domains of EnvC, ActS, and NlpD are also sometimes referred to as degenerate “dLytM” domains in recognition that they lack the enzymatic activity present in the original protein from which they are named (7). Activators are themselves typically autoinhibited and are activated at specific times and places to regulate amidase activity.

One of the best understood amidase activation systems is the FtsEX–EnvC complex. FtsEX is a Type VII ABC transporter (18–20) that belongs to the same protein superfamily as MacB (19), LolCDE (21–23), BceAB (24), and HrtBA (25). During cell division, and after recruitment to the septal Z-ring (26), ATP binding and hydrolysis by FtsEX drives conformational changes in EnvC that facilitate binding and activation of AmiA and AmiB in the periplasm (17, 27). A structure of *E. coli* EnvC bound to the periplasmic domains of FtsX shows that EnvC is itself autoinhibited by the presence of a helix (the restraining arm) that blocks access to the amidase-binding groove in the EnvC LytM domain (17). Conformational change in FtsEX–EnvC is predicted to displace the restraining arm providing access for the amidase to bind the LytM domain (17); however, molecular details of how activator binding induces amidase activation remain poorly understood.

Here, we present a crystal structure of the AmiA peptidoglycan amidase in its as-isolated “resting” state and an activated form of the AmiB enzymatic domain bound to the EnvC LytM domain. Our structures show precisely how activator binding displaces the autoinhibitory helix of the amidase to allow substrate access and reorganizes the active site to promote peptidoglycan hydrolase activity.

## Results

### A Structure of AmiA Defines Its Active Site and Regulatory Domain.

**Fig. 1.**
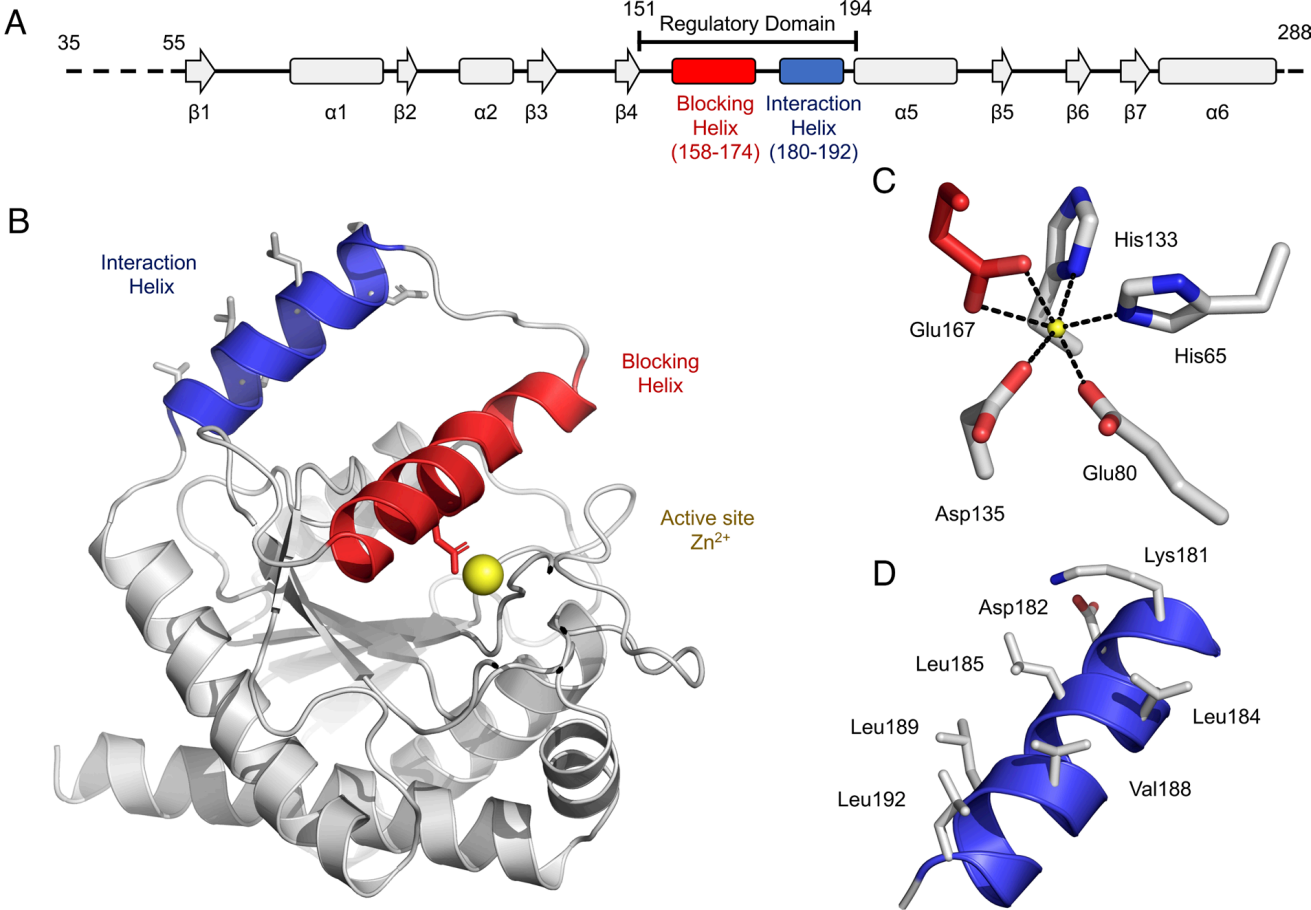
Structure of *E. coli* AmiA determined by X-ray crystallography. (*A*) Secondary structure diagram for AmiA. (*B*) 3D structure of AmiA from the 2.3 Å crystal structure. (*C*) Active-site zinc and ligating residues. (*D*) Solvent-facing hydrophobic residues on the AmiA interaction helix.

We determined a crystal structure of *E. coli* AmiA using X-ray crystallography. Crystals of AmiA diffract to a resolution of 2.4 Å and contain two molecules per asymmetric unit. Full diffraction data and refinement statistics are given in *SI Appendix*, Table S1. The secondary structure of AmiA is diagrammed in Fig. 1*A*, and a representative monomer from the structure is shown in Fig. 1*B*. As expected from its amino acid similarity, the overall fold of AmiA is very similar to previous structures of *Bartonella henselae* AmiB (11) and *E. coli* AmiC (5), including the active site (*SI Appendix*, Fig. S1). Each AmiA monomer consists of a single globular domain with a six-membered beta sheet and six alpha helices (Fig. 1 *A* and *B*). The AmiA active site is composed of a single zinc atom that is held in place by two histidine residues (His65 and His133), an aspartate (Asp135), and two glutamates (Glu80 and Glu167) (Fig. 1*C*). Similar to AmiB (11) and AmiC (5), the AmiA active-site zinc is not accessible to peptidoglycan substrates due to the presence of an alpha helix that occludes the active site; we term this feature the “blocking helix” (Fig. 1 *A* and *B*, red). As demonstrated in subsequent sections, the blocking helix has a role in autoinhibiting the activity of AmiA and forms part of a larger regulatory domain (residues 151–194) which includes a second alpha helix that constitutes the binding site for EnvC. We define the latter feature as the “interaction helix” (Fig. 1 *A* and *C*, blue). The interaction helix consists of residues 180-192 and stands conspicuously proud from the rest of the molecule. The interaction helix is also notable for possessing five solvent-facing hydrophobic residues (Leu184, Leu185, Val188, Leu189, Leu192) and is the only feature for which we identify meaningful conformational differences between the two AmiA molecules observed in the crystal structure (Fig. 1*D*). In one chain, the interaction helix is well defined, while in the other, the corresponding electron density is smeared out, consistent with thermal motion. We further assessed the dynamics of AmiA by plotting the B-factors from each monomer against sequence and performing molecular dynamics simulations (*SI Appendix*, Fig. S2). Both the experimental data and simulations consistently show that the regulatory domain is much more dynamic than the rest of the protein. The overall structure of AmiA is consistent with an autoinhibited form of the enzyme in which the active-site zinc is occluded by the blocking helix, while a potential protein interaction site remains exposed to solvent ready for activation.

### Mutational Analysis of the AmiA Autoinhibitory Domain.

To test whether the regulatory domain maintains AmiA in an autoinhibited state, we made mutations that are predicted to relieve autoinhibition and monitored bacterial viability and detergent sensitivity when these variants were expressed in the periplasm. Expression of wild-type AmiA does not significantly disrupt viability or detergent sensitivity of *E. coli*. However, expression of AmiA variants that lack the regulatory domain causes a reduction in bacterial viability and increases bacterial sensitivity to detergent (*SI Appendix*, Fig. S3*A*). This was the case for three distinct AmiA regulatory domain deletion constructs, each engineered with different regulatory domain deletions (*SI Appendix*, Fig. S3*B*). In addition to the regulatory domain deletions, we also tested single-amino acid substitutions in Glu167 which is located on the blocking helix and, in the crystal structure, is directly ligated to the active-site zinc. The equivalent residue has previously been shown to be a key residue in maintaining autoinhibition for both AmiB (11) and AmiC (6). Mutations of Glu167 to glutamine or lysine are modestly effective in relieving AmiA autoinhibition as judged by the detergent sensitivity of strains expressing these variants in the periplasm (*SI Appendix*, Fig. S4*A*). Molecular dynamics simulations of AmiA and AmiA Glu167 mutants provide useful context to these experiments, showing that the blocking helix fluctuates between bound and free positions in the mutants, but remains locked firmly in place for the wild type (*SI Appendix*, Fig. S4 *B* and *C*). These observations are consistent with a role for the blocking helix in AmiA autoinhibition, with Glu167 forming a “latch” that anchors the blocking helix to the active-site zinc.

### Mutations in the Interaction Helix Break the Interaction between AmiA and Its Activator.

**Fig. 2.**
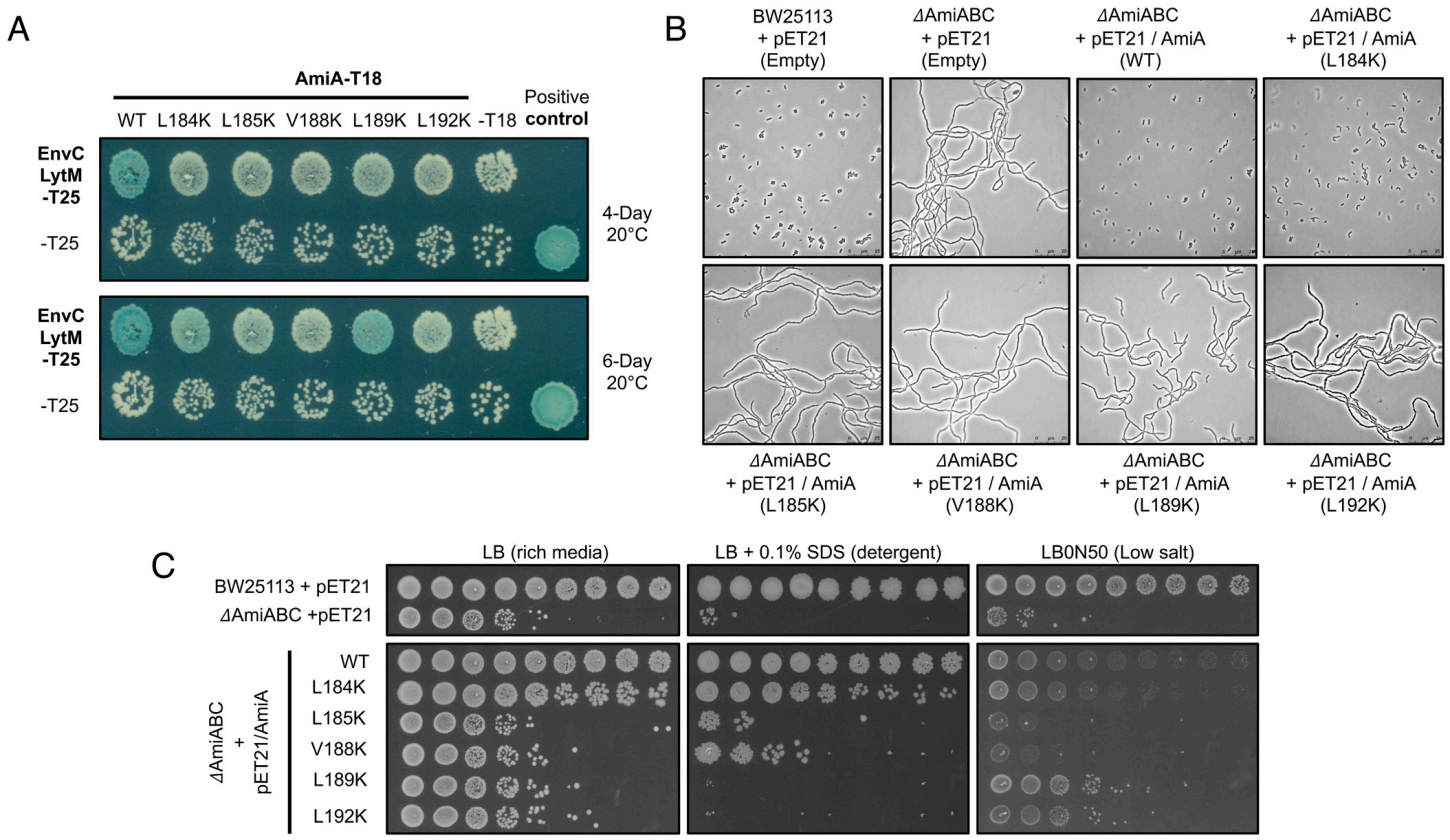
Testing the importance of the AmiA interaction helix. (*A*) Bacterial 2-hybrid experiment testing the interaction between the EnvC LytM domain and AmiA for the wild-type proteins and five AmiA single amino acid variants. The top and bottom panels show the same agar plate photographed after 4 or 6 d at 20 °C. (*B*). Phase-contrast images of bacterial cultures after overnight growth. BW25113 indicates the “wild-type” *E. coli* strain and *ΔamiABC* indicates a triple-deletion knockout strain. Strains carry either an empty vector (pET21) or full-length AmiA construct (pET21/AmiA) encoding either the wild-type protein or indicated variant (*C*). Viability assays. Cultures are spotted as a series dilution, from left to right, starting with a culture adjusted to OD 1 with a 10-fold dilution at each step. LB agar reports on general viability, while SDS and LBON50 (low salt media) report on outer membrane integrity and sensitivity to osmotic challenge, respectively.

We next turned our attention to the function of the interaction helix. Based on the structure of AmiA, we hypothesized that the solvent-facing hydrophobic residues presented along the face of the interaction helix mediate binding to the cognate activator (EnvC). Using a bacterial 2-hybrid experiment, we assessed the interaction between AmiA and the EnvC LytM domain after introducing point mutations into the interaction helix. Wild-type AmiA binds strongly to the EnvC LytM domain, but lysine substitutions of any of the solvent-facing hydrophobics completely disrupt the interaction (Fig. 2*A*). When left for a longer period, some variants did show detectable signs of interaction—although these were significantly weaker than for the wild type consistent with partial disruption of the interaction (Fig. 2*A*). To control for the possibility that these mutations might destabilize the amidase, or differentially affect expression levels, we also ran an SDS-PAGE gel to detect the expression of each variant under identical bacterial growth conditions; all mutants were detected at the correct molecular weight with similar intensity across the gel (*SI Appendix*, Fig. S5).

To further analyze the effect of interaction helix mutations in vitro, we coexpressed a subset of AmiA variants alongside the His-tagged EnvC LytM domain and assessed the stability of the complex using copurification (*SI Appendix*, Fig. S6 *A* and *B*). Consistent with the bacterial 2-hybrid data, both AmiA L184K and L185K copurify in lower yield than the wild type, and the AmiA L188K variant does not interact at all, even though all AmiA variants are highly expressed in comparison to the LytM domain (*SI Appendix*, Fig. S6*C*). We therefore conclude that the AmiA interacts with EnvC via its surface-exposed interaction helix.

### Mutations in the Interaction Helix Block the Function of AmiA In Vivo.

To further dissect the function of AmiA, and the role of the interaction helix, we established a multiamidase knockout strain of *E. coli* BW25113 that could be complemented by AmiA or AmiA variants (*SI Appendix*, Fig. S7). As expected from a previous triple-knockout study (3), both the cell division defect and detergent susceptibility phenotypes of the triple-amidase mutants can be corrected by expression of AmiA from a plasmid. Using this system, we tested various AmiA mutants for their ability to rescue these defects in the Δ*amiABC* background using the empty vector as a control. We used phase-contrast microscopy to inspect cells for the chaining phenotype (Fig. 2*B*), and viability on detergent agar as an indicator of cell envelope integrity (Fig. 2*C*). Four of the five AmiA variants (L185K, V188K, L189K, and L192K) and the empty vector control were highly chained and detergent sensitive, while the wild-type AmiA rescued both defects and appeared otherwise indistinguishable from the parental strain. The fifth mutant, L184K, was only modestly chained with detergent sensitivity close to wild type. These data confirm the importance of the solvent-facing residues in the interaction helix for in vivo functionality of AmiA and are consistent with roles for these residues in interactions with EnvC.

### Structure of the AmiB Hydrolase Domain Bound to the Amidase-Activating EnvC LytM Domain.

To better understand the molecular basis for activation of the FtsEX–EnvC-dependent amidases, we sought to determine a crystal structure of an amidase bound to its cognate LytM domain. Our strategy was to identify well-expressed and stable amidase activator pairs and screen for crystallization using robotics. Using copurification experiments, we first demonstrated that AmiA could be successfully purified with isolated LytM domain of EnvC (*SI Appendix*, Fig. S8). We also showed that AmiA does not copurify with either the full-length EnvC protein or an EnvC construct lacking the coiled coil, consistent with EnvC being autoinhibited by the presence of the EnvC restraining arm in these constructs. These experiments complement previous work showing copurification of the EnvC periplasmic domain with AmiB, and bacterial 2-hybrid assays confirming this pattern of interactions for AmiA and AmiB in *E. coli* (17). The experiment also confirms that the same autoinhibition mechanism that regulates EnvC’s activation of AmiB applies to AmiA.

Screening activator/amidase pairs from multiple organisms, we identified several well-expressed amidase hydrolytic domains that copurified with their cognate EnvC LytM domains. This included both AmiA and AmiB constructs, the latter of which were cloned without their N-terminal “AmiN” domain. After extensive crystallization trials, we were ultimately successful in solving a crystal structure of the AmiB hydrolytic domain bound to the EnvC LytM domain using proteins from *Citrobacter rodentium*.

**Fig. 3.**
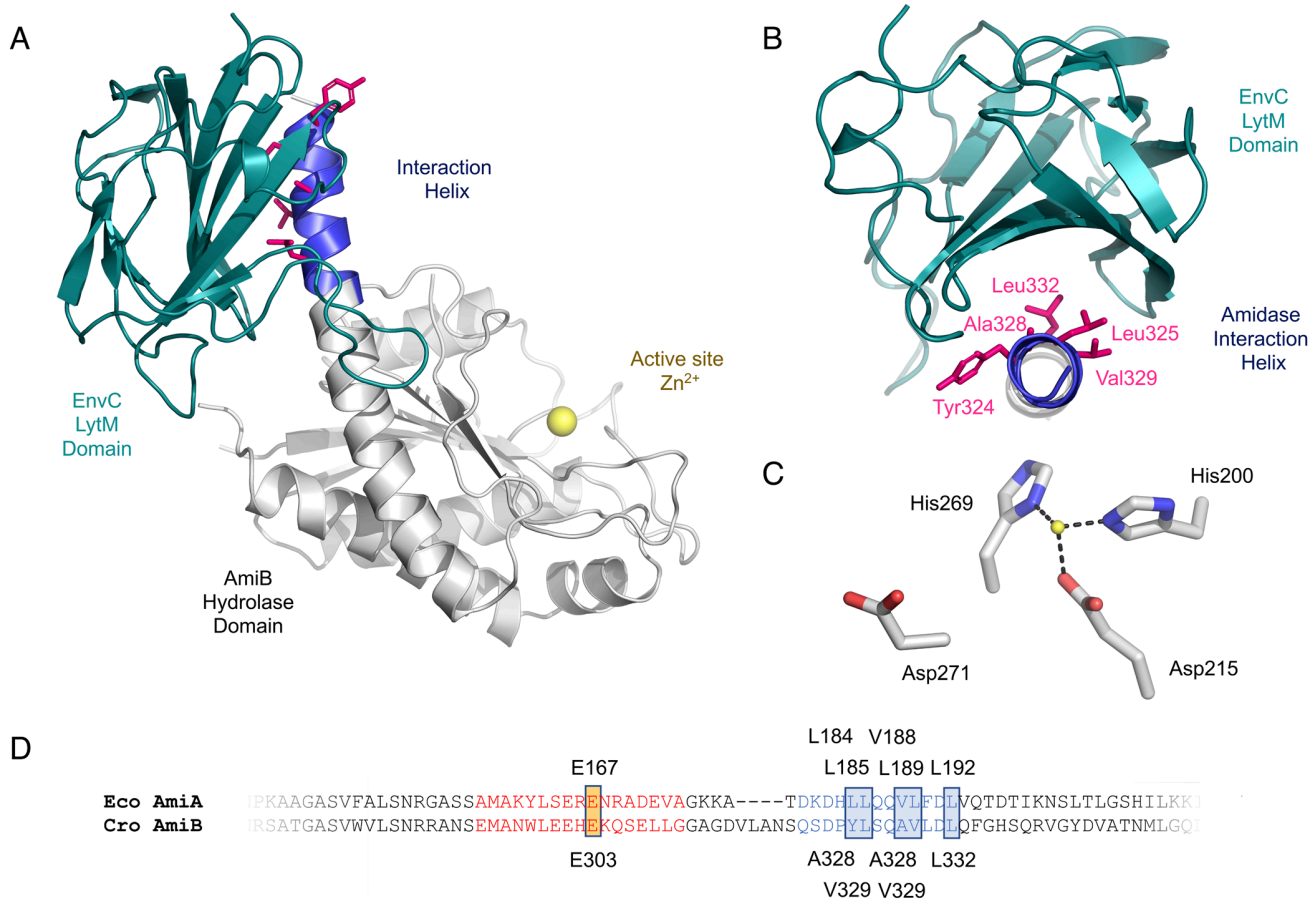
Structure of the amidase–activator complex. (*A*) 3.4 Å crystal structure of the AmiB enzymatic domain from *C. rodentium* bound to the cognate LytM activation domain of EnvC. (*B*) Close-up view of AmiB–EnvC–LytM interface. (*C*) Close-up of the active-site ligands in the AmiB–EnvC–LytM complex. (*D*) Sequence alignment between *E. coli* AmiA and *C. rodentium* AmiB centered over the regulatory domain. Amino acids corresponding to the blocking helix and interaction helix are shown in red and blue, respectively. Key residues are highlighted to assist identification of functionally equivalent residues.

The 3.4 Å structure of the AmiB hydrolytic domain bound to the EnvC LytM domain is shown in Fig. 3. Inspecting the architecture of the complex (Fig. 3*A*), three observations are immediately apparent. First, the EnvC LytM domain is bound directly to the amidase interaction helix, with the latter’s hydrophobic residues all pointing directly into the LytM groove (Fig. 3*B*). Second, the amidase regulatory domain has a very different conformation in the EnvC-bound structure such that the interaction helix is contiguous with helix 5 and the blocking helix is displaced from the active site (Fig. 3*A*). Finally, the active-site zinc is ligated by three residues rather than the five (Fig. 3*C*) due to the absence of the blocking helix glutamate (Glu167 in AmiA) and dissociation of one of the aspartates (Asp271 in AmiB, equivalent to Asp135 in AmiA). An alignment of AmiA and AmiB regulatory domain sequences is provided in Fig. 3*D* to assist the reader in matching equivalent residues. The structure confirms the predicted importance of the surface-facing hydrophobic residues along the interaction helix and confirms binding-induced conformational change as a mechanism for amidase activation.

### The Amidase’s Interaction Helix Binds in the Same Groove as the EnvC Restraining Arm.

**Fig. 4.**
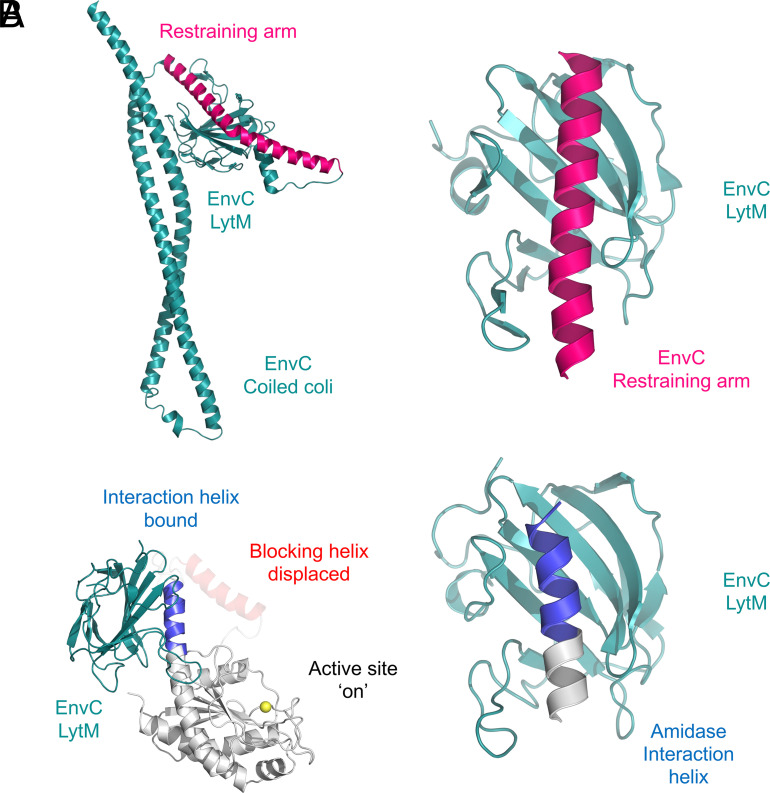
The amidase interaction helix binds in the same groove as the EnvC restraining arm. (*A*) Structure of EnvC showing the autoinhibitory restraining arm (*pink*) bound within its LytM domain (*teal*). A close-up view is shown to the right. (*B*) Structure of the activated amidase bound by the activating EnvC LytM domain. The displaced blocking helix (*red*) was too disordered to build in the crystal structure of the complex (most likely due to high mobility), but is shown here in a semi-transparent form to indicate a feasible position given the observed location of the (well ordered) interaction helix (*blue*). A close-up view of the amidase interaction helix bound in the LytM groove is shown to the right for comparison with the restraining-arm-bound LytM domain immediately above.

In a crystal structure of full-length EnvC bound to the two periplasmic domains of FtsX, it was noted that the LytM domain is occupied by a long helix (the “restraining arm”) that blocks access to the amidase-binding groove (17). This led to the proposal that the restraining arm would need to be displaced by a conformational change to allow amidase to bind and be activated. The conformational change is expected to be driven by ATP binding and hydrolysis by FtsEX and propagated through the coiled coil of EnvC. The structure of the EnvC–LytM AmiB complex shows that the amidase interaction helix binds within the same groove as the restraining arm, lending further support for this mechanism (Fig. 4 *A* and *B*).

The interface of the AmiB–EnvC complex is governed by interactions between the exterior hydrophobic residues of the amidase and residues lining the interior of the EnvC LytM groove. Consistent with the structure, several contact residues inside the LytM groove have previously been identified as important for amidase activation in *E. coli* EnvC (7). Additionally, the activator–amidase structure further identifies a distinctive loop (*C. rodentium* EnvC residues G319–G330) that contacts residues located *between* the interaction helix and helix 5. The extended loop wraps around the residues located between the interaction helix and helix 5, causing them to form a single, continuous, helical element (Fig. 4*B*). Taken together, the two amidase structures capture a significant conformational change in the regulatory domain as the N-terminal end of interaction helix is prised away from the enzymatic domain by the binding of EnvC. The knock-on effect of this levering motion is to pull the sequence-neighboring blocking helix away from the zinc exposing the active site for peptidoglycan binding.

### Reorganization of the Activated Zinc Site in the EnvC-Bound Amidase.

In addition to the dislocation of the blocking helix, which is tied to dissociation of Glu167/Glu303 from the active-site zinc, we also observed displacement of Asp135/Asp271 (Fig. 3*C*). Consequently, the amidase zinc site is surrounded by five residues in the resting state (Fig. 1*C*) but only three in the activated (EnvC LytM bound) state (Fig. 3*C*). In the EnvC LytM AmiB costructure, the angles between neighboring ligating residues and the zinc are all close to 109°, suggesting a tetrahedral coordination state in which the fourth position remains open for substrate binding and catalysis.

Inspecting the density surrounding the zinc, a low-occupancy ligand is present at the fourth position of the coordination sphere. The ligand is consistent with a sugar molecule in chair conformation (*SI Appendix*, Fig. S9). No sugars were used in crystallizing the complex and thus this molecule seems to have been copurified during protein production. The sugar could be a product of peptidoglycan hydrolysis; however, due to modest resolution (3.4 Å) and partial occupancy, we have not yet formally identified this molecule. We anticipate that future high-resolution studies may be able to resolve this ligand, and perhaps characterize further natural substrates, reaction products, or even inhibitors, bound to the amidase.

## Discussion

Peptidoglycan amidases are key hydrolytic enzymes that are needed to break the peptidoglycan layer during cell division to allow for separation of daughter cells. Here, we described two crystal structures that capture both the active and inactive states of the amidase, showing precisely how division-associated peptidoglycan hydrolases are activated by their interaction with a cognate partner, EnvC. We first described the crystal structure of an isolated *E. coli* amidase, AmiA, at 2.4 Å resolution. The structure reveals an inactive form of the enzyme where the active-site zinc is blocked by an autoinhibitory helix, with a solvent-facing helix that forms the binding site for its cognate activator (Fig. 1). We then showed the importance of the interaction helix using site-directed mutagenesis. Mutations in the interaction helix disrupt interaction with EnvC and prevent activation of the amidase in vivo (Fig. 2). A structure of AmiB bound to the EnvC LytM domain further establishes the interaction helix as the EnvC-binding site and reveals the activation mechanism; the amidase interaction helix docks inside of an EnvC surface groove, forcing conformational changes in the neighboring autoinhibitory helix that expose the active site (Fig. 3). Displacement of the blocking helix not only makes the active site accessible to substrates, but also reconfigures the ligands surrounding the active-site zinc. Finally, we show that the groove in the LytM domain of EnvC that forms the amidase-binding site is the same groove that is blocked by the EnvC restraining arm (Fig. 4). The structures show that autoinhibition is a feature of both the amidase and the activator, and that the interaction between the activator and amidase involves substantial conformational changes.

**Fig. 5.**
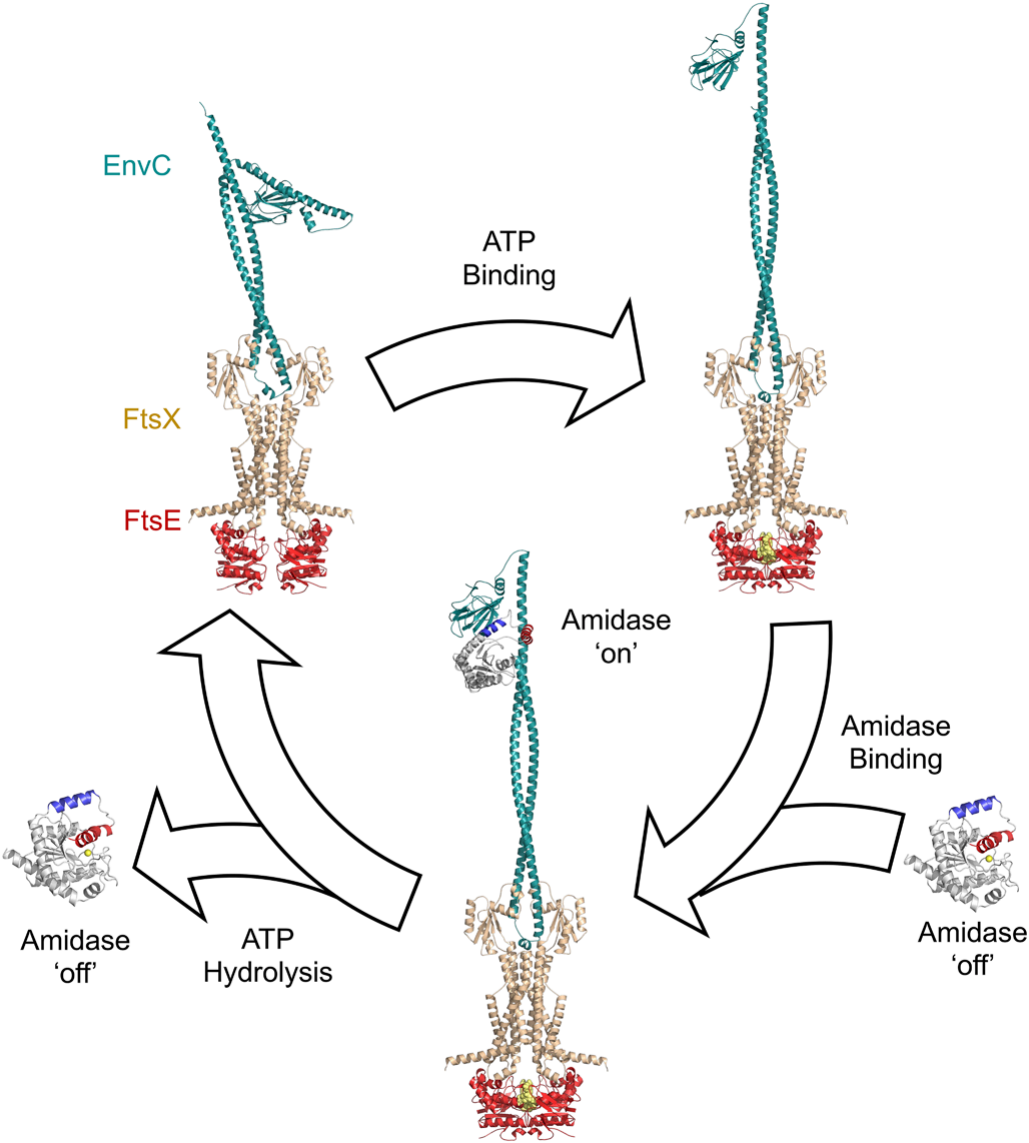
Mechanism of amidase activation by FtsEX–EnvC. Full details are given in the *Discussion*. Theoretical models are constructed using a combination of Alphafold predictions (28, 29) and experimentally determined crystal structures [6TPI (17), 8C2O, and 8C0J].

An updated mechanism for amidase activation in the FtsEX–EnvC–AmiA system is presented in Fig. 5. During periods of inactivity, both the amidase and the FtsEX–EnvC complex are autoinhibited (Fig. 5, *Top Left*): AmiA is autoinhibited by its blocking helix which prevents the binding of peptidoglycan and the FtsEX–EnvC complex is autoinhibited by the restraining arm which prevents recruitment of the amidase to the LytM domain. Upon ATP binding to FtsEX–EnvC, a long-range conformational change is transmitted through EnvC, freeing the LytM domain from the restraining arm and exposing the amidase-binding site (Fig. 5, *Top Right*). Binding of the amidase to the EnvC LytM domain relies upon the amidase interaction helix, which binds in the same location from which the restraining arm was displaced (Fig. 5, *Bottom*). Upon binding, the amidase undergoes an induced conformational change in which the blocking helix is displaced from the active site. Rearrangement of the ligands surrounding the active-site zinc leads to peptidoglycan amidase activity. Finally, ATP hydrolysis allows the system to reset; the amidase is released and EnvC restraining arm returns to the LytM groove (Fig. 5, *Top Left*). A near-identical mechanism likely operates for FtsEX–EnvC–AmiB, although in that case the amidase may additionally be prelocated at the division site by interactions between the N-terminal “AmiN” domain and the peptidoglycan layer (4–7). For AmiA, which lacks an AmiN domain, localization to the division site most likely relies on interactions between FtsEX–EnvC or other components of the division machinery. A key feature of this proposed mechanism is that the amidase is only briefly activated since the eventual hydrolysis of ATP by FtsEX returns the complex to the inactive autoinhibited state. Once the amidase is released, it is rapidly autoinhibited by the blocking helix returning to the peptidoglycan binding groove.

LytM domains are widespread among amidase activators (12), with predicted LytM domains in both NlpD (30) (the activator for AmiC) and ActS (12, 14) (formerly known as YgeR—an activator of AmiC, AmiA, and AmiB) of *E. coli*. The interaction described here for EnvC and its cognate amidases (AmiA and AmiB) may well serve as a useful model for understanding these interactions.

The apparent redundancy between division-associated amidases and the large number of murein hydrolase activators raises the question of why such complexity is required. This is especially true for the FtsEX–EnvC–AmiA/AmiB system since the gram-positive equivalent, FtsEX–PcsB, operates without separate amidases and instead uses an EnvC-like protein (PcsB) that has its own peptidoglycan hydrolase activity (31, 32). Maintaining amidases in several different parts of the gram-negative cell envelope may be advantageous for separating the peptidoglycan layer while coordinating invagination of the outer membrane, as suggested for NlpD–AmiC (30). Overlapping specificity of amidases and activators may be useful under different environmental stress conditions as has been suggested for ActS (33).

In summary, we have determined the structures of two peptidoglycan amidases (AmiA and AmiB) in autoinhibited and activated states and relate these to their wider regulation through interactions with a cognate activator, the LytM domain of FtsEX–EnvC. The structures reveal near-atomic details of the conformational changes in the amidase that are required for activation of peptidoglycan hydrolysis including displacement of the autoinhibitory helix, and rearrangement of the sidechains that surround the active-site zinc. Our data significantly advance our understanding of a key event in bacterial cell division (breakage of the peptidoglycan layer) and provide fascinating molecular insights into the conformational changes that regulate amidase activity.

## Methods

A full set of methods are given in *SI Appendix*. In brief, structures of AmiA and the complex between the AmiB enzymatic domain and the EnvC LytM domain were determined using X-ray crystallography using software from CCP4 suite (34) with molecular replacement probes generated by Alphafold (28, 29). Coordinates and structure factors have been deposited with the protein data bank (accession codes **8C2O** and **8C0J**). Bacterial viability and detergent susceptibility were assessed by spotting out bacterial cultures in 10-fold series dilution on LB agar or LB agar supplemented with 0.1 % (w/v) SDS. All strains carry a plasmid providing ampicillin resistance as a selection marker and agar was supplemented with 50 μg/mL ampicillin and 1 mM IPTG. MICs were determined in microbroth culture using LB containing 50 μg/mL ampicillin and 1 mM IPTG. Bacterial 2-hybrid experiments used the BACTH system (35). The wild-type *E. coli* BW25113 and single-amidase knockout strains were obtained from the Keio collection (36). The double- and triple-amidase knockout strains were produced in the same background using Genebridges gene deletion kit (37). Phase-contrast microscopy was performed after overnight growth in LB containing 1mM IPTG and 50 μg/mL ampicillin. Molecular dynamics simulations used Gromacs (38) with the Charm forcefield (39). Structural figures were produced with Pymol (40).

## Supplementary Material

Appendix 01 (PDF)

## Data Availability

Crystal structure coordinates and structure factors data have been deposited in Protein Data Bank (8C2O (41) is the structure of *E. coli* AmiA and 8C0J (42) is the structure of the AmiB enzymatic domain bound to the EnvC LytM domain.
