# Supplementary material for: Activator-induced conformational changes regulate division-associated peptidoglycan amidases
Source: Proc Natl Acad Sci U S A. 2023 Jun 5;120(24):e2302580120. doi: 10.1073/pnas.2302580120 (PMC10268282; doi:10.1073/pnas.2302580120)
Supplement: Supplementary file 1 — Appendix 01 (PDF) [file pnas.2302580120.sapp.pdf]

Supplemental Information

Activator-induced conformational changes regulate division-associated peptidoglycan amidases

Jonathan Cook†, Tyler C. Baverstock†, Martin B.L. McAndrew, David I. Roper, Phillip J. Stansfeld, Allister Crow*

Schedule of Supplemental Information

- Table S1: Data collection and refinement statistics.
- Table S2: Plasmids used in this study.
- Figure S1: Comparison of *E. coli* AmiA with structures of *Bartonella henselae* AmiB and *E. coli* AmiC.
- Figure S2: Flexibility in AmiA.
- Figure S3: Overexpression of AmiA constructs lacking their regulatory domains causes an outer membrane defect in *E. coli*.
- Figure S4: Mutational analysis of AmiA Glu167.
- Figure S5: Control experiment showing expression of AmiA variants.
- Figure S6: Co-expression and co-purification of AmiA interaction helix variants with the EnvC LytM domain.
- Figure S7: Characterization of cell envelope integrity for single, double and triple amidase deletion strains in a common genetic background.
- Figure S8: Co-expression and purification experiments for AmiA and three EnvC variants.
- Figure S9: Electron density for an unidentified ligand at the AmiB active site.
- Methods
- Supplemental References

Table S1: Data collection and refinement statistics

	AmiA<br>8C2O	EnvC-LytM:AmiB<br>Complex<br>8C0J
**Data collection**		
Beam line	Diamond I04-1	Diamond I04
Wavelength (Å)	0.97950	0.95373
**Crystal parameters**		
Space group	$P\ 2\ 2_1\ 2_1$	$I\ 4_1\ 3\ 2$
Unit cell dimensions (Å)	59.5, 73.9, 115.6	237.4, 237.4, 237.4
Unit cell angles (°)	90, 90, 90	90, 90, 90
**Reflection data**[*]		
Resolution range (Å)	59.51-2.35 (2.43-2.35)	59.34-3.38 (3.65-3.38)
Unique reflections	21,955 (2,121)	16,294 (3,305)
R_{pim}	0.050 (0.506)	0.088 (0.686)
$I/\sigma(I)$	10.4 (1.5)	9.9 (1.6)
$CC_{1/2}$	0.999 (0.662)	0.998 (0.939)
Completeness (%)	100 (100)	100 (100)
Multiplicity	12.6 (12.1)	78.2 (78.9)
Wilson B ($Å^2$)	51	81
**Refinement**[†]		
Resolution (Å)	59.60 - 2.35	50.00 - 3.38
Number of reflections	20,819	15,286
$R_{overall}$	0.177	0.249
R_{free}	0.236	0.291
Rms (bond lengths) (Å)	0.007	0.008
Rms (bond angles) (°)	1.43	1.41
**Model B-factors**		
Proteins ($Å^2$)	50, 60	110, 123, 142
Zn ($Å^2$)	42, 43	95, 139
Waters ($Å^2$)	43	-
**Ramachandran statistics**[‡]		
Favoured (%)	97.6	91.9
Allowed (%)	2.4	7.9
Outlier (%)	0	0.2

Values in parentheses indicate the highest resolution bin.
Refinement statistics are from Refmac.
Ramachandran statistics as reported by Rampage.

Table S2: Plasmids used in this study.

Plasmid	Vector type	Contents	Tag	Figure
pTB1 034	pET21a	sol AmiA (35-289)	C term HIS	1
pTB1 022	pETDuet-1	EnvC (35-419) plus AmiA (35-289)	N term HIS	S8
pTB1 023	pETDuet-1	EnvC (222-419) plus AmiA (35-289)	N term HIS	S8
pTB1 016 *	pETDuet-1	EnvC (277-419) plus AmiA (35-289)	N term HIS on EnvC (277-419)	S8, S6
pCRoAmiBenzEnvCact	pETDuet-1	Citrobacter LytM (284-420) plus enzAmiB (190-442)	N term HIS	3
pTB1 028	pET21a	full AmiA	no tag	S3, S4, S5
pTB1 029	pET21a	full AmiB	no tag	S5
pTB1 030	pET21a	full AmiC	no tag	S5
pTB1 050	pET21a	full AmiA Δreg-1	no tag	S3
pTB1 055	pET21a	full AmiA Δreg-2	no tag	S3
pTB1 056	pET21a	full AmiA Δ-reg-3	no tag	S3
pTB1 011 *	pUT18C	sol AmiA (35-289) wt	-T18	2, S4
pJC6 622	pUT18C	sol AmiA L184K	-T18	2, S4
pJC6 624	pUT18C	sol AmiA L185K	-T18	2, S4
pJC6 627	pUT18C	sol AmiA V188K	-T18	2, S4
pJC6 629	pUT18C	sol AmiA L189K	-T18	2, S4
pJC6 645	pUT18C	sol AmiA L192K	-T18	2, S4
pJC6 257 *	pK N T25	EnvC LytM (278-419)	-T25	2, S4
pJC6 620	pUC	full AmiA		
pJC6 659	pUC	full AmiA E167K		
pJC6 698	pUC	full AmiA E167Q		
pJC6 882	pUC	full AmiA L184K		
pJC6 885	pUC	full AmiA L185K		
pJC6 888	pUC	full AmiA V188K		
pJC6 893	pUC	full AmiA L189K		

pJC6 894	pUC	full AmiA L192K		
pJC6 689	pET21a	full AmiA E167K	no tag	S4
pJC6 709	pET21a	full AmiA E167Q	no tag	S4
pJC6 898	pET21a	full AmiA L184K	no tag	S4
pJC6 899	pET21a	full AmiA L185K	no tag	S4
pJC6 901	pET21a	full AmiA V188K	no tag	S4
pJC6 908	pET21a	full AmiA L189K	no tag	S4
pJC6 909	pET21a	full AmiA L192K	no tag	S4
pJC7 054	pETDuet-1	EnvC (277-419) plus AmiA (35-289) (L184K)	N-term His-tag on EnvC (277-419)	S6
pJC7 056	pETDuet-1	EnvC (277-419) plus AmiA (35-289) (L185K)	N-term His-tag on EnvC (277-419)	S6
pJC7 058	pETDuet-1	EnvC (277-419) plus AmiA (35-289) (L188K)	N-term His-tag on EnvC (277-419)	S6

* Indicated vectors from Cook et al 2020 (1).

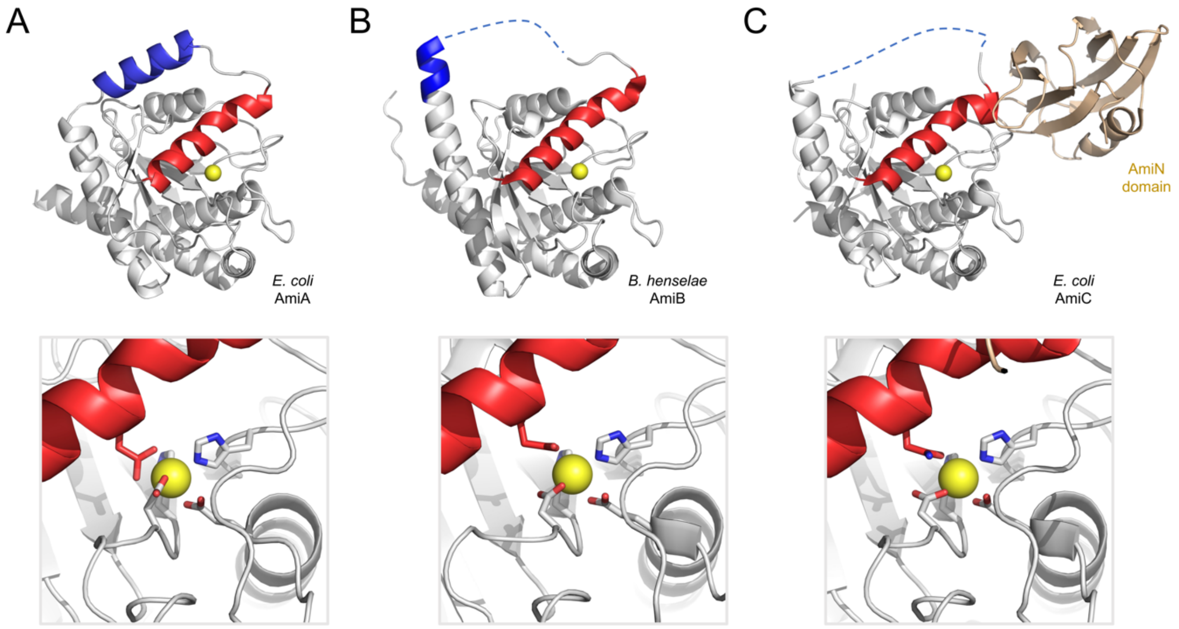

Figure S1: Comparison of *E. coli* AmiA with structures of *Bartonella henselae* AmiB and *E. coli* AmiC. (A) Structure *E. coli* AmiA presented here (pdb entry 8C2O). Red indicates the autoinhibitory blocking helix and interaction helix in blue. The other structures are coloured similarly. (B) Structure of AmiB from *Bartonella henselae* (2) (3NE8 chain A). The N-terminal domain is not present. (C) Structure of *E. coli* AmiC (3) (4BIN chain A). A short linker between the AmiN domain and the enzymatic domain (residues 152-162) is hidden for clarity. Close-up views of the zinc active site are shown below the structures. An unmodelled loop in both AmiB and AmiC is shown as a dotted line. Enzymatic domains of AmiB and AmiC can be superposed on AmiA with RMSD values of 0.76 Å and 0.51 Å respectively.

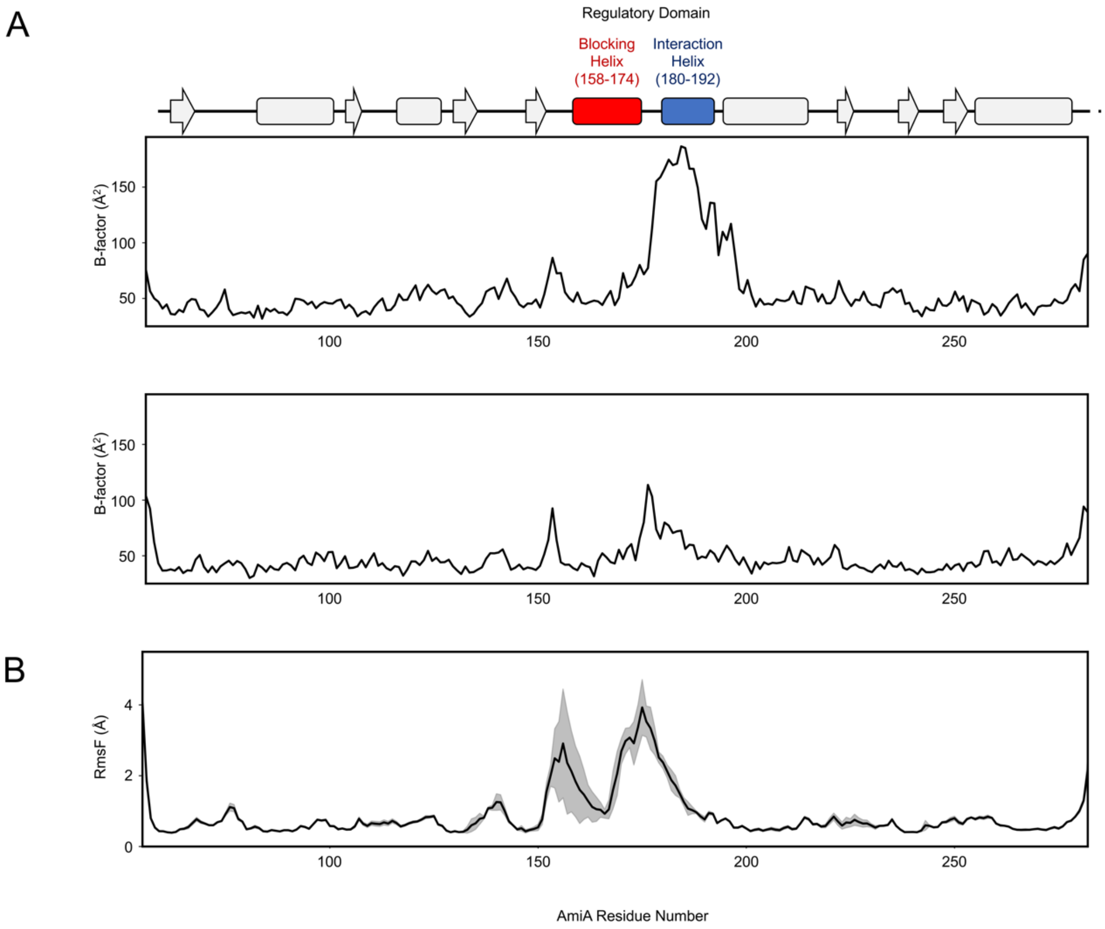

Figure S2: Flexibility in AmiA. (**A**) B-factor plots for AmiA showing high mobility in the Regulatory domain. (**B**) Plot of RmsF (Root-mean-square Fluctuations) over the course of a 500 ns simulation of AmiA.

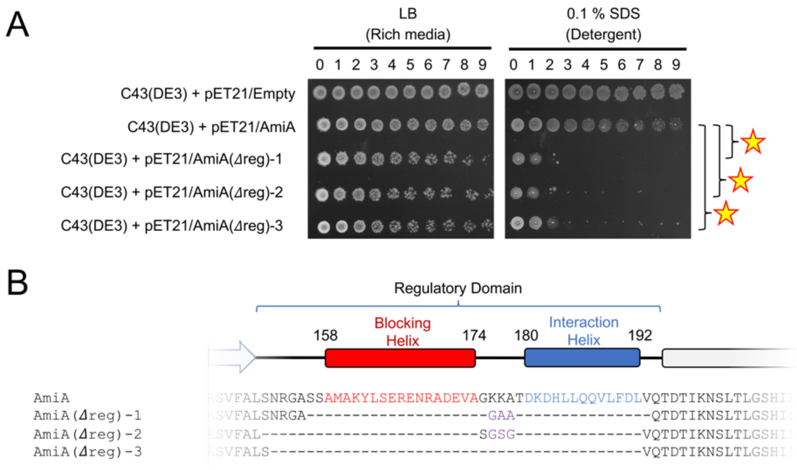

Figure S3: Overexpression of AmiA constructs lacking their regulatory domains causes an outer membrane defect in *E. coli*. (**A**) Viability assays for *E. coli* C43(DE3) carrying either an empty vector, wild type AmiA, or one of three AmiA constructs lacking the regulatory domain. Stars indicate significant differences in detergent sensitivity. (**B**) Amino acid sequence alignment for AmiA and three constructs engineered to lack the regulatory domain. The three constructs differ in the design of the linker replacing the regulatory domain deletion.

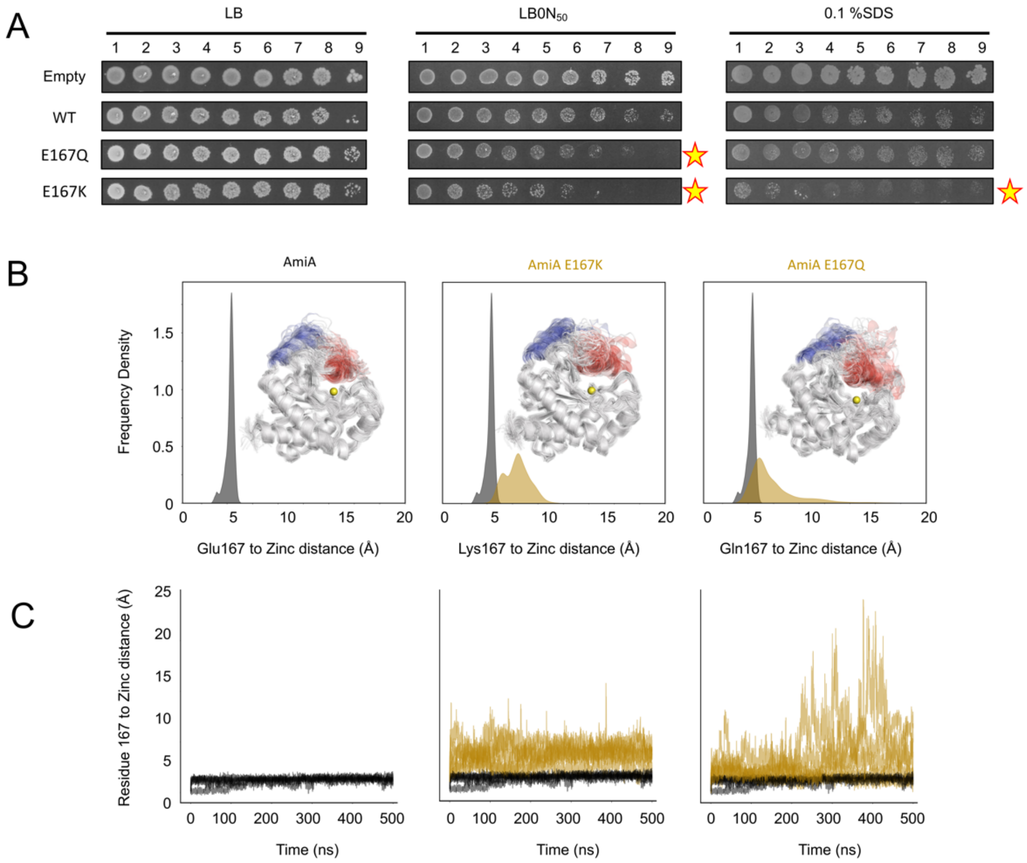

Figure S4: Mutational analysis of AmiA Glu167. (**A**) Viability assays for *E. coli* expressing either wild type AmiA or the indicated Glu167 variant (E167Q, E167K). Cultures were adjusted to OD600 =1 and series diluted using 10-fold steps. Cells were then spotted on LB, LB0N50 or 0.1 % SDS to test viability, osmotic sensitivity and detergent sensitiviy. (**B**) Histograms showing the distribution of distances between the Glu167 C-alpha atom and the active site zinc over the course of five 500 ns simulations of AmiA, AmiA E167Q and E167K. Structural ensembles taken from the simulation are shown inset. (**C**) Plots of the distance between the Glu167 and active site zinc over the course of the simulations.

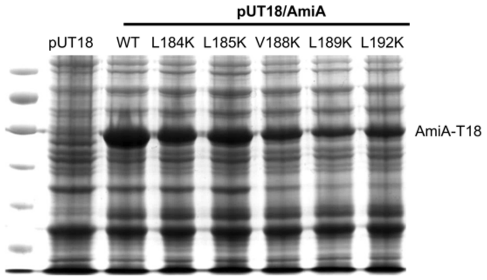

Figure S5: Control experiment showing expression of AmiA variants. The AmiA-T18 fusions used in the bacterial 2-hybrid were expressed in *E. coli* C43 (DE3) and whole cell lysates subjected to SDS PAGE. Lysates from cells carrying the empty pUT18 vector were used as a negative control.

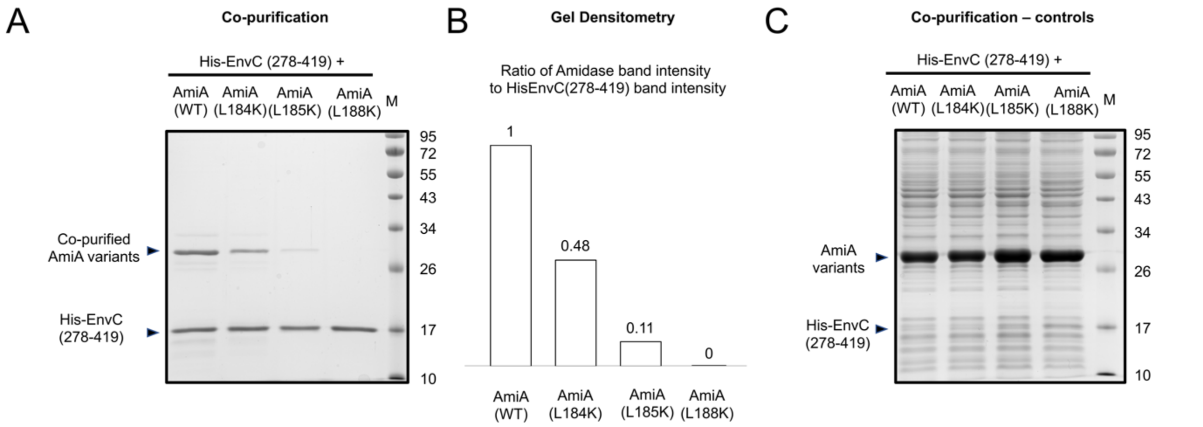

Figure S6: Co-expression and co-purification of AmiA interaction helix variants with the EnvC LytM domain. (A) SDS-PAGE gel showing eluted EnvC-LytM:Amidase complexes after Ni-IMAC purification. Only the EnvC LytM domain is His-tagged, so co-purification of non-tagged amidase variants are dependent on sufficient affinity for the EnvC LytM domain. (B) Gel densitometry of the lefthand gel showing the ratio of amidase band intensity to that of the LytM domain. The ratio of band intensity for the wild type is close to 1:1, while interaction helix variants co-purify with significantly less amidase. There is no sign of an interaction between EnvC LytM and the L188K variant. (C) SDS-PAGE gel showing cleared cell lysates from *E. coli* co-expressing each AmiA variants alongside the His-EnvC LytM domain. These are samples used as input for the Ni-IMAC purification and serve as controls for the expression and stability of the AmiA variants. All the amidases are stably expressed and located at high concentration on the soluble fraction.

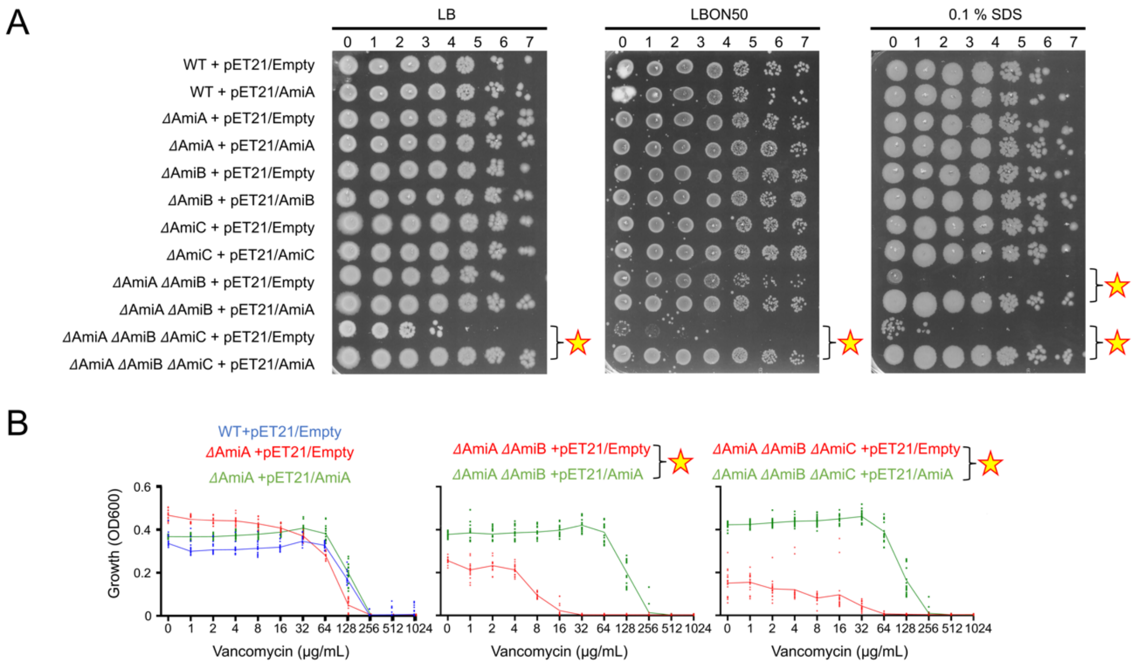

Figure S7: Characterization of cell envelope integrity for single, double and triple amidase deletion strains in a common genetic background. (**A**) Viability of *E. coli* strains with different amidase sets on regular, low salt and high detergent LB agar. WT indicates the wild type (parental) strain, *E. coli* BW25113. Single, double, and triple amidase knockout strains are derived from the parental strain. All strains carry a pET21a based vector (providing ampicillin resistance) which is either empty or carrying the indicated *E. coli* amidase gene. The number of 10-fold serial dilutions from an initial OD 600 1.0 culture is indicated above each agar plate. Stars indicate significant differences in viability between strains complemented by AmiA verses an empty vector. (**B**) Antibiotic susceptibility assays for the wild type strain (BW2511, *left*) and AmiA deletion strain (*Δ*AmiA, *left*), double amidase deletion (*Δ*AmiA *Δ*AmiB, *centre*) and triple amidase deletion (*Δ*AmiA *Δ*AmiB *Δ*AmiC, *right*). Each strain carries either an empty plasmid (pET21a) or AmiA expressing plasmid (pET/AmiA). We find that viability of most single amidase knockouts are similar to the parental strain, but that the uncomplemented triple knockout (*Δ*AmiA *Δ*AmiB *Δ*AmiC +pET21/Empty) shows reduced viability, osmotic sensitivity and susceptibility to SDS and vancomycin (an antibiotic that would not usually pass the outer membrane barrier in *E. coli*). Remarkably, these phenotypes can be almost completely reversed by modest expression of AmiA from a plasmid (*Δ*AmiA *Δ*AmiB *Δ*AmiC +pET21/AmiA).

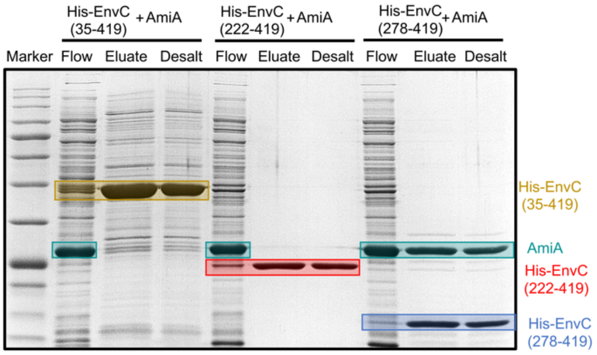

Figure S8: Co-expression and purification experiments for AmiA and three EnvC variants. SDS-PAGE gel showing fractions from three Ni-IMAC purifications. Co-expressed AmiA and His-tagged EnvC variants are indicated above the gel. Samples from the column Flowthrough, a high-imidazole Elution, and a sample taken after removing the imidazole using desalting column are shown for each purification. Untagged AmiA is visible in the flow through of all three experiments, showing it is co-expressed in each case. Co-purification of AmiA was only observed for the His-tagged EnvC-LytM protein.

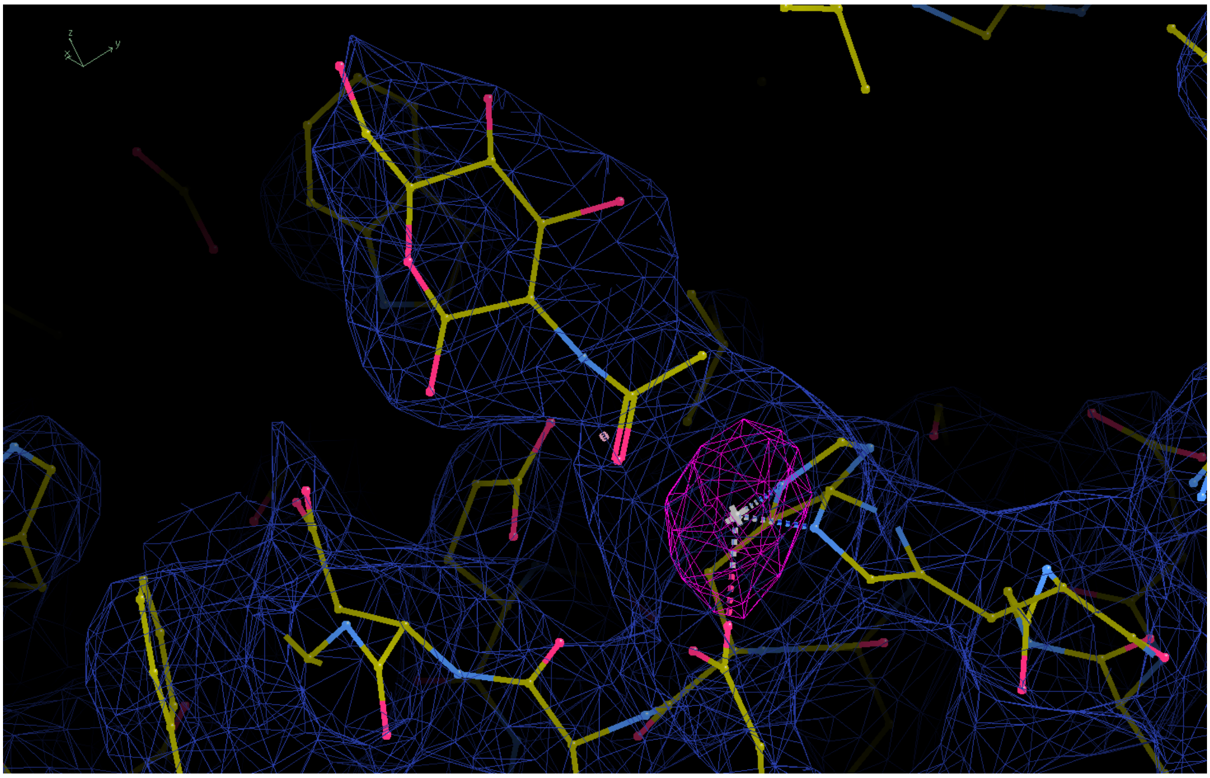

Figure S9: Electron density for an unidentified ligand at the AmiB active site. The standard weighted $2m|F_o|-|F_c|$ map is contoured at 1.25 σ. An anomalous difference map confirming the presence of Zinc is shown in pink, contoured at 5 σ. Figure generated with Coot. Extra electron density opposite the active site zinc most likely indicates the presence of a sugar molecule. A molecule of N-acetyl glucosamine (NAG) is shown built into the residual density. In the deposited coordinates, the sugar is omitted because we have not yet been able to confirm its identity by orthogonal methods. A second monomer of AmiB in the asymmetric unit does not contain the sugar molecule.

Methods

Cloning

All constructs used in this study are listed in **Table S2.** All DNA sequences were confirmed by DNA sequencing (Genewiz). Genes encoding AmiA, AmiB and AmiC were amplified by PCR from *E. coli* DH5α genomic DNA. The products were digested with NdeI and XhoI restriction enzymes and ligated into the similarly digested pET21a. AmiA lacking its signal sequence was generated by PCR and cloned into pET21a via NdeI/XhoI to obtain a C-terminal His tag. This AmiA gene was also ligated into the 3' site of pETDuet-1 via NdeI/XhoI followed by sections of EnvC into the 5' site via BamHI/EcoRI. For bacterial 2-hybrid experiments, AmiA was further subcloned into pUT18C via BamHI/EcoRI.

For co-expression of the amidase-activator, the LytM domain of EnvC (284-420) and the enzymatic domain of AmiB (190-422) from *Citrobacter rodentium* were synthesised by Genscript (with codon optimisation) and engineered into the first and second multiple cloning sites of pETDuet-1 via the BamHI/NotI and NdeI/XhoI sites.

For testing AmiA constructs lacking the regulatory domain, DNA encoding full length AmiA (including an encoded secretion signal) were synthesised by Genscript in pET21a via NdeI/XhoI sites. TB1050 expresses a periplasm-directed AmiA construct in which residues 156-193 (inclusive) were replaced with the amino acid sequence GAA. Tb1055 expresses a periplasm-directed AmiA construct in which residues 152-192 (inclusive) were replaced with the amino acid sequence GSG, and TB1056 expresses a periplasm-directed AmiA construct in which residues 152-192 (inclusive) were removed without replacement.

For site-directed mutagenesis of AmiA, an XbaI/EcoRI fragment encoding the full length AmiA gene was subcloned from pTB1028 into a pUC18 vector and point mutations introduced using the Quikchange Protocol. The mutated genes were subcloned back into pET21a via NdeI/XhoI for expression studies, and also into the Bacterial 2 Hybrid System vectors via the BamHI and EcoRI sites after an intermediate PCR to ensure that the open-reading frame was maintained.

Protein expression and purification

For production of AmiA, plasmid pTB1034 (AmiA with C-terminal His tag) was transformed into *E. coli* C43 (DE3), grown in 2YT at 30 °C to an OD_{600nm} of 0.6 when 1 mM IPTG was

added. After 18 hours further growth, cells were harvested by centrifugation (6,000 x*g*), the cell pellets were resuspended in wash buffer (50 mM HEPES pH 7.2, 300 mM NaCl, 35 mM imidazole) and lysed by 3 passages through an Avestin C3 homogeniser (15,000 psi). The lysate was centrifuged at 30,000 x *g* for 30 min at 6 °C to remove cellular debris and the supernatant loaded onto an immobilised Ni-affinity column pre-equilibrated with the wash buffer. Bound proteins were washed with 25 column volumes of wash buffer before elution in 50 mM HEPES pH 7.2, 300 mM NaCl, 250 mM imidazole. Protein samples were buffer exchanged into 20 mM HEPES pH 7.2, 300 mM NaCl and stored at -80 °C.

For production of the EnvC AmiB complex, N-terminally His-tagged EnvC LytM domain was co-expressed with the enzymatic domain of AmiB from a pETDuet-based plasmid as above, except that the pH of the buffers was maintained at pH 8.0. Purified complexes were then buffer exchanged into 20 mM HEPES pH 8.0, 350 mM NaCl and concentrated to 5.5 mg/ml using a centrifugal filter (Amicon 30 kDa nominal molecular weight cut-off).

Interaction of AmiA with EnvC variants

Co-expression constructs were transformed into *E. coli* C43, grown in 2YT at 30 °C to an OD 600nm of 0.6 when 1 mM IPTG was added. After 18 hours further growth, cells were harvested by centrifugation (6,000 x *g*), the cell pellets were resuspended in wash buffer (50 mM HEPES pH 7.2, 300 mM NaCl, 35 mM imidazole) and lysed using an Avestin C3 homogeniser (15,000 psi). The lysate was centrifuged at 30,000 x *g* for 30 min at 6 °C to remove cellular debris and the supernatant loaded onto an immobilised Ni-affinity column pre-equilibrated with the wash buffer. Bound proteins were washed with 25 column volumes of wash buffer before elution in 50 mM HEPES pH 7.2, 300 mM NaCl, 250 mM imidazole. Excess salt and imidazole was then removed using a PD10 desalting column equilibrated in 20 mM HEPS pH7.2, 150 mM NaCl. For SDS-PAGE analysis, all samples were diluted 10-fold and mixed with a denaturing loading buffer before adding to the gel.

For co-expression and co-purification of AmiA interaction helix variants, experiments were performed as above using expression from pETDuet vectors and co-purification via Ni-IMAC resin. The eluates and cleared lysates were subjected to SDS-PAGE. The quantity of His-tagged LytM domain in the eluate was assessed using gel densitometry with the Biorad Image Lab software and a new gel was run adjusting the volumes loaded so that the amount of LytM domain was similar across lanes. The gel was then further analysed to obtain the band intensity of the LytM domain and any co-purified amidase and a ratio calculated.

Crystallisation

Crystallisation screens were performed using a Formulatrix NT8 crystallisation robot. *E. coli* AmiA was crystallised using a crystallisation reagent composed of (1.4 M tri-sodium citrate and 0.1 M HEPES pH 7.5) and the AmiB-EnvC lytM domain complex (from *Citrobacter rodentium*) was crystallised using (0.8 M Sodium phosphate monobasic monohydrate, 0.8 M Potassium phosphate monobasic, 0.1 M Sodium HEPES, pH 7.5). Both crystallisations used the sitting drop vapour diffusion method with 1 uL drops composed of 666 nL protein solution and 333 nL crystallisation reagent) equilibrated against an 80 μL reservoir of the reagent alone in MRC 2-drop plates. Protein crystals were harvested in litholoops and flash-frozen in liquid nitrogen using standard cryo-crystallography methods.

Structure determination

X-ray diffraction experiments were performed using remote data collection at the Diamond Light Source UK synchrotron. Diffraction images were indexed and integrated using Dials via the synchrotrons data processing pipeline and subsequently scaled inhouse using Aimless (4). Further processing used programs from the CCP4 suite (5). Initial phase determination used the molecular replacement method implemented in Phaser (6). For AmiA, we used an initial structure prediction generated using the Alphafold prediction algorithm (7) as implemented in Colabfold (8). For the amidase-activator complex, we used our experimentally determined model of AmiA and the LytM domain of *E. coli* EnvC extracted from PDB entry 6TPI - a structure of EnvC bound to the periplasmic domains of FtsX (1). Models were completed using alternating rounds of model re-building with Coot (9) and refinement with Refmac (10). Non-crystallographic symmetry restraints were applied in both cases. Refinement of the AmiB-EnvC complex additionally used secondary restraints generated using ProSMART (11). Validation used tools in Procheck (12) and Coot (9) to assess geometry, and Rampage (13) to assess the distribution of backbone angles in the Ramachandran plot. The presence of zinc was confirmed using X-ray fluorescence and calculation of anomalous difference maps. Coordinates and structure factors have been deposited at the protein data bank.

Construction of amidase knockout strains

Single knockout *E. coli* strains lacking *amiA* and *amiB* were taken directly from the Keio collection. These strains are both derivatives of the parent strain, *E. coli* BW25113, and have a kanamycin cassette on the chromosome in place of the amidase-encoding gene (BW25113 *ΔamiA::Kan* and BW25113 *ΔamiB::Kan*). The *amiAB* double knockout (BW25113 *ΔamiA*

ΔamiB::Kan) was constructed by removing the kanamycin cassette from the BW25113*ΔamiA::Kan* strain using FLP-FRT recombination, and then using lambda red recombination (14) (as implemented in the Genebridges gene deletion kit) to re-insert a kanamycin cassette into the *amiB* gene. The triple deletion was constructed similarly, by first removing the kanamycin cassette from the BW25113 *ΔamiA ΔamiB::Kan* double knockout strain, and then re-inserting a kanamycin resistance cassette into the *amiC* gene. All strains were checked for kanamycin resistance and verified using PCR amplification of the three gene sites using primers on either side of each genetic locus.

***E. coli* viability assays on agar plates**

E. coli strain BW25113, variations of this parental strain with different genes 'knocked out' and the C43 strain were transformed with plasmids carrying either the wild type AmiA, AmiB, AmiC or variant AmiA. Cells were grown in LB supplemented with 50 µg/ml ampicillin to OD_{600} = 1.0. Each culture was 10-fold serially diluted in LB supplemented with 50 µg/ml ampicillin and 1 mM IPTG. 3 µl of each dilution was spotted onto LB agar containing 50 µg/ml ampicillin, 1 mM IPTG and with/without 0.1% SDS. Culture was also spotted onto LBON50 agar (LB with no salt, diluted 2-fold with water) with 50 µg/ml ampicillin and 1 mM IPTG. Plates were incubated overnight at 37 °C and then imaged using an Epson scanner. Images were uniformly contrast-adjusted and converted to greyscale.

Vancomycin MIC determination

Minimum inhibitory concentrations were determined in LB supplemented with 50 µg/ml ampicillin and 1 mM IPTG. Each set of vancomycin concentrations was generated by 2-fold serial dilutions. Experiments were conducted in 96-well plates with a final volume of 200 µl in each well, all of which were seeded with 5 µl of starter culture that was preadjusted to OD_{600} = 0.01 by dilution of seed cultures that were grown to between 0.6 and 1.0 (OD_{600}). After 18 hours of growth at 37 °C, plates were read in a MultiSkan Sky plate reader (Thermo Scientific) using the absorbance at 600 nm wavelength. MICs are presented as the median of several determinations (typically, twenty-four MIC measurements consisting of three biological repeats with eight technical repeats for each).

Bacterial 2-hybrid protein-protein interaction studies

The Bacterial Adenylate Cyclase Two Hybrid (BACTH) system was used to perform bacterial 2-hybrid experiments (15). 2 ng of each of two complimentary plasmids were co-transformed into *E. coli* BTH101 cells and grown overnight at 30 °C in LB (50 µg/ml ampicillin, 25 µg/ml kanamycin). 5 µl was spotted onto LB agar plates containing 50 µg/ml ampicillin, 25 µg/ml kanamycin, 40 µg/ml X-gal and 0.5 mM IPTG and grown at 20 °C for ~64 hr. The negative control was a pair of empty pUT18 and pKT25 vectors. The positive control was a pair of plasmids containing a leucine zipper which dimerises. The presence of blue colonies indicated that the expressed proteins had interacted.

Phase Contrast Microscopy

Triple knockout *E. coli* ΔAmiABC cells carrying pET21a-based plasmids encoding AmiA variants were grown overnight in LB supplemented with 50 µg/ml ampicillin and 1mM IPTG at 37 °C. A sample of each was diluted with LB and spotted onto an agarose-coated glass slide for immediate observation. Images were collected using a Leica Microsystems Model TL LED microscope.

Molecular Dynamics Simulations

All simulations were performed using Gromacs 2021 using the charmm36m forcefield (16, 17). Simulated proteins were appropriately protonated, centred, and solvated with TIP3P water in a rhombic dodecahedron box with 0.15 M NaCl. Systems were energy minimized using a steepest descent algorithm to 1000 kJ mol^{-1} nm^{-1} before equilibration for 1 ns with 1000 x 1000 x 1000 kJ mol^{-1} nm^{2} position restraints on all protein atoms (excluding hydrogens). Post equilibration, five production repeats were generated and simulated unrestrained for 500 ns, each with different starting velocities. A 2 fs timestep was applied to an NPT ensemble with either an isotropic C-rescale barostat for production simulations or an isotropic Berendesen barostat for equilibration simulations at 1 bar and V-rescale temperature coupling where protein and solvent were individually coupled (18–20). Electrostatic terms were described using PME with a cut-off of 1.2 nm while van der Waals interactions were shifted between 1 and 1.2 nm. Hydrogen bonds were constrained with LINCS (21) and SETTLE (22) was used to constrain the water bond angles and distances. Analysis was performed using Gromacs and MDAnalysis (23). PyMOL was used for visualisation (24).

Supplemental References

1. J. Cook, *et al.*, Insights into bacterial cell division from a structure of EnvC bound to the FtsX periplasmic domain. *Proc. Natl. Acad. Sci. U.S.A.* **117**, 28355–28365 (2020).

2. D. C. Yang, K. Tan, A. Joachimiak, T. G. Bernhardt, A conformational switch controls cell wall-remodelling enzymes required for bacterial cell division: Control of cell wall-remodelling enzymes. *Molecular Microbiology* **85**, 768–781 (2012).

3. M. Rocaboy, *et al.*, The crystal structure of the cell division amidase AmiC reveals the fold of the AMIN domain, a new peptidoglycan binding domain: Crystal structure of AmiC of *Escherichia coli*. *Molecular Microbiology*, n/a-n/a (2013).

4. P. R. Evans, G. N. Murshudov, How good are my data and what is the resolution? *Acta Crystallogr D Biol Crystallogr* **69**, 1204–1214 (2013).

5. M. D. Winn, *et al.*, Overview of the *CCP* 4 suite and current developments. *Acta Crystallogr D Biol Crystallogr* **67**, 235–242 (2011).

6. A. J. McCoy, *et al.*, *Phaser* crystallographic software. *J Appl Crystallogr* **40**, 658–674 (2007).

7. J. Jumper, *et al.*, Highly accurate protein structure prediction with AlphaFold. *Nature* **596**, 583–589 (2021).

8. M. Mirdita, *et al.*, ColabFold: making protein folding accessible to all. *Nat Methods* **19**, 679–682 (2022).

9. P. Emsley, B. Lohkamp, W. G. Scott, K. Cowtan, Features and development of *Coot*. *Acta Crystallogr D Biol Crystallogr* **66**, 486–501 (2010).

10. G. N. Murshudov, *et al.*, *REFMAC* 5 for the refinement of macromolecular crystal structures. *Acta Crystallogr D Biol Crystallogr* **67**, 355–367 (2011).

11. R. A. Nicholls, M. Fischer, S. McNicholas, G. N. Murshudov, Conformation-independent structural comparison of macromolecules with *ProSMART*. *Acta Crystallogr D Biol Crystallogr* **70**, 2487–2499 (2014).

12. R. A. Laskowski, M. W. MacArthur, D. S. Moss, J. M. Thornton, PROCHECK: a program to check the stereochemical quality of protein structures. *J Appl Crystallogr* **26**, 283–291 (1993).

13. S. C. Lovell, *et al.*, Structure validation by Cα geometry: ϕ,ψ and Cβ deviation. *Proteins* **50**, 437–450 (2003).

14. Y. Zhang, F. Buchholz, J. P. P. Muyrers, A. F. Stewart, A new logic for DNA engineering using recombination in Escherichia coli. *Nat Genet* **20**, 123–128 (1998).

15. G. Karimova, J. Pidoux, A. Ullmann, D. Ladant, A bacterial two-hybrid system based on a reconstituted signal transduction pathway. *Proc. Natl. Acad. Sci. U.S.A.* **95**, 5752–5756 (1998).

16. M. J. Abraham, *et al.*, GROMACS: High performance molecular simulations through multi-level parallelism from laptops to supercomputers. *SoftwareX* **1–2**, 19–25 (2015).

17. J. Huang, *et al.*, CHARMM36m: an improved force field for folded and intrinsically disordered proteins. *Nat Methods* **14**, 71–73 (2017).

18. M. Bernetti, G. Bussi, Pressure control using stochastic cell rescaling. *J. Chem. Phys.* **153**, 114107 (2020).

19. G. Bussi, D. Donadio, M. Parrinello, Canonical sampling through velocity rescaling. *The Journal of Chemical Physics* **126**, 014101 (2007).

20. H. J. C. Berendsen, J. P. M. Postma, W. F. van Gunsteren, A. DiNola, J. R. Haak, Molecular dynamics with coupling to an external bath. *The Journal of Chemical Physics* **81**, 3684–3690 (1984).

21. B. Hess, H. Bekker, H. J. C. Berendsen, J. G. E. M. Fraaije, LINCS: A linear constraint solver for molecular simulations. *J. Comput. Chem.* **18**, 1463–1472 (1997).

22. S. Miyamoto, P. A. Kollman, Settle: An analytical version of the SHAKE and RATTLE algorithm for rigid water models. *J. Comput. Chem.* **13**, 952–962 (1992).

23. N. Michaud-Agrawal, E. J. Denning, T. B. Woolf, O. Beckstein, MDAnalysis: A toolkit for the analysis of molecular dynamics simulations. *J. Comput. Chem.* **32**, 2319–2327 (2011).

24. Schrödinger, LLC, The PyMOL Molecular Graphics System, Version 1.8 (2015).
